# Understanding the Modulatory Effects of Cannabidiol on Alzheimer’s Disease

**DOI:** 10.3390/brainsci11091211

**Published:** 2021-09-14

**Authors:** Yinyi Xiong, Chae-Seok Lim

**Affiliations:** 1 Department of Pharmacology, Wonkwang University School of Medicine, Iksan 54538, Korea; x1992889@gmail.com; 2 Department of Rehabilitation, Affiliated Hospital of Jiujiang University, Jiujiang 332000, China

**Keywords:** Alzheimer’s disease, cannabidiol, cannabinoid receptor, endocannabinoid system, amyloid β, phosphorylated tau

## Abstract

Alzheimer’s disease (AD), the most common neurodegenerative disease, is characterized by progressive cognitive impairment. The deposition of amyloid beta (Aβ) and hyperphosphorylated tau is considered the hallmark of AD pathology. Many therapeutic approaches such as Food and Drug Administration-approved cholinesterase inhibitors and *N*–methyl–D–aspartate receptor antagonists have been used to intervene in AD pathology. However, current therapies only provide limited symptomatic relief and are ineffective in preventing AD progression. Cannabidiol (CBD), a phytocannabinoid devoid of psychoactive responses, provides neuroprotective effects through both cannabinoid and noncannabinoid receptors. Recent studies using an AD mouse model have suggested that CBD can reverse cognitive deficits along with Aβ-induced neuroinflammatory, oxidative responses, and neuronal death. Furthermore, CBD can reduce the accumulation of Aβ and hyperphosphorylation of tau, suggesting the possibility of delaying AD progression. Particularly, the noncannabinoid receptor, peroxisome proliferator-activated receptor gamma, has been suggested to be involved in multiple functions of CBD. Therefore, understanding the underlying mechanisms of CBD is necessary for intervening in AD pathology in depth and for the translation of preclinical studies into clinical settings. In this review, we summarize recent studies on the effect of CBD in AD and suggest problems to be overcome for the therapeutic use of CBD.

## 1. Introduction

Alzheimer’s disease (AD), which is the most common neurodegenerative disease, is characterized by progressive cognitive deficits before the normal aging process. The global disruption of patients’ cognitive abilities such as short-term memory loss, spatial disorientation, executive dysfunction, and inability of facial recognition accelerates the weakening of the physical state and aggravates the burden on their caregivers and society. The prevalence of AD is predicted to increase in the near future because of increased life expectancy.

AD is characterized as either familial or sporadic. Regarding the age of onset, familial AD, which results from mutations in genes encoding amyloid precursor protein (APP) or presenilin1/2 (PS1/2), the catalytic component of the γ-secretase complex usually occurs earlier than sporadic AD. However, although sporadic AD accounts for up to 95% of cases, the information on the cause of sporadic AD is lacking. Nevertheless, the well-known pathological hallmarks—amyloid plaques, neurofibrillary tangles, and synapse loss—can be observed across both types of AD. Amyloid plaques are extracellular deposits of amyloid beta (Aβ), which are generated by an aberrant sequential cleavage of APP. To date, compelling evidence has supported the causal relationship between the elevation of Aβ and AD progression in various in vitro or in vivo models. Mouse models with one or multiple mutations in APP or PS1 can develop partial histopathological and behavioral AD features and are invaluable in pathological and pharmaceutical research areas. Although it is difficult to recapitulate the pathology of hyperphosphorylated tau, which contributes to the formation of neurofibrillary tangles in APP-transgenic AD mice, a close link between Aβ accumulation and tau pathology has been reported in induced pluripotent stem cell-derived neurons and three dimensional (3D) organoids of AD [1,2]. Moreover, a murine model carrying both mutant tau and APP transgenes suggested that Aβ accumulation unidirectionally influences the appearance of hyperphosphorylated tau and the development of neurofibrillary tangles in AD [3]. Overall, this evidence indicates that the Aβ-initiated tau-dependent pathway leads to synaptic dysfunction and memory deficits, whereas loss of phosphorylated tau can protect against Aβ toxicity [4,5].

**Figure 1:**
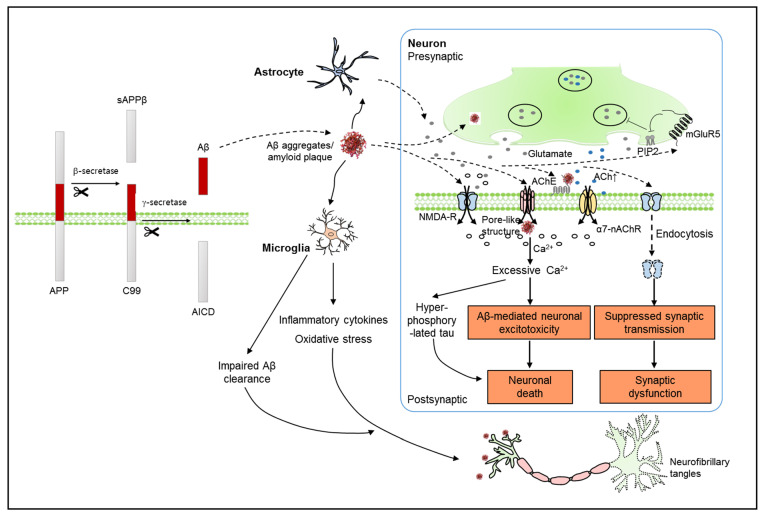
Schematic representation of the amyloid hypothesis for AD pathology. Amyloid beta (Aβ), generated by the aberrant sequential cleavage of APP, aggregates to form extracellular amyloid plaques. As a trigger, Aβ induces toxic amounts of Ca^2+^ influx through the activation of postsynaptic NMDA receptors or pore-like structures generated by Aβ. Aβ inhibits the activity of AChE and increases ACh levels in the synaptic cleft, contributing to excessive intracellular Ca^2+^ concentration. High levels of intracellular Ca^2+^ mediate a series of downstream signaling pathways related to neuronal excitotoxicity. Aβ-initiated hyperphosphorylated tau results in neuronal death. On the other hand, Aβ mediates synaptic dysfunction by inducing endocytosis of postsynaptic NMDA receptors or presynaptic mGluR5-mediated depletion of PIP_2_, which decreases neurotransmission. Aβ-induced activation of astrocytes and microglia contributes to neuronal degeneration in AD through excitotoxicity, neuroinflammation, oxidative stress, and impaired Aβ clearance.

The role of Aβ in AD pathology is a trigger for various subsequent amyloid cascades that ultimately contribute to cognitive impairment (Figure 1). The major cascades involved in Aβ-mediated neurotoxicity include excitotoxicity, oxidative stress, and apoptosis. Aβ increases glutamate release from neurons and astrocytes. Increased extracellular glutamate excessively activates both synaptic and extrasynaptic *N*-methyl-D-aspartate (NMDA) receptors, leading to intense transient influx of Ca^2+^ and activation of signaling pathways responsible for neuronal dysfunction and cell death [6,7]. In addition to alteration of Ca^2+^ homeostasis through NMDA receptors, Aβ oligomers can destabilize the plasma membrane and make pores, leading to toxic levels of Ca^2+^ influx [8,9]. Moreover, Aβ can promote endocytosis of NMDA receptors to suppress synaptic transmission [10,11]. It has been reported that Aβ suppresses synaptic transmission through presynaptic metabotropic glutamate receptor 5 (mGluR5)-mediated depletion of phosphatidylinositol-4,5-bisphosphate (PIP_2_) [12]. In addition, the failure of Aβ clearance accelerates the development of AD. Although early microglial recruitment can benefit Aβ clearance, persistent microglial activation causes microglia to lose the anti-inflammatory phenotype and increase the release of inflammatory cytokines, reactive oxygen species (ROS), and chemokines and thereby, further supplements Aβ production and toxicity [13]. It is noteworthy that autophagy deficiency can exacerbate intraneuronal Aβ accumulation by inhibiting Aβ secretion and affect memory loss [14].

Because of the potential role of reduced cholinergic neurotransmission and NMDA receptor-mediated excitotoxicity in cognitive deficits, currently available Food and Drug Administration (FDA)-approved drugs for AD are compounds that facilitate cholinergic neurotransmission or antagonize NMDA receptor activity. However, these therapies only provide small symptomatic benefit without any neuroprotective effect against neurodegeneration [15,16]. In addition, based on the crucial involvement of oxidative stress and neuroinflammation in AD progression, researchers have explored the therapeutic potential of antioxidants and nonsteroidal anti-inflammatory drugs in AD [13,17]. Unfortunately, many studies have indicated that neither of them is recommended as a common treatment for AD because they increase the risk of mortality and gastrointestinal problems with limited efficacy [16,18].

Based on the amyloid hypothesis, scientists explored the feasibility of antiamyloid therapy. Beta- and gamma (γ)-secretase are responsible for Aβ production from APP. However, inhibition of γ-secretase causes severe side effects because of the extensive functions of γ-secretase in our body [19,20] and development of a β-secretase inhibitor is challenging [21]. In addition, another antiamyloid therapy exhibited limited efficacy and was associated with development of meningoencephalitis [22,23]. However, patients who developed higher levels of antibodies against Aβ showed relatively better cognitive performance [24]. Although aducanumab (Aduhelm) against Aβ [25] was recently approved by the US FDA, it has many limitations due to brain swelling and tiny brain bleeds, no effect on the middle and late phases of AD, and too high a cost to be effective. Therefore, antiamyloid immunotherapy has not greatly altered the antibody therapy for AD.

Overall, the strategy that targets a single pathway seems unrealistic for delaying or ceasing AD progression. Currently, neuroprotective drugs that simultaneously target multiple AD pathologies are being studied to mitigate the detrimental effects of Aβ and finally arrest AD progression. In this context, the cannabinoid and endocannabinoid system (ECS), which regulates various pathological processes related to AD, has attracted interest from researchers [26]. In this review, we summarize recent studies on the effect of CBD, a phytocannabinoid, in Alzheimer’s disease and suggest problems to be overcome for the therapeutic use of CBD.

## 2. Alzheimer’s Disease and the Endocannabinoid System (ECS)

**Figure 2:**
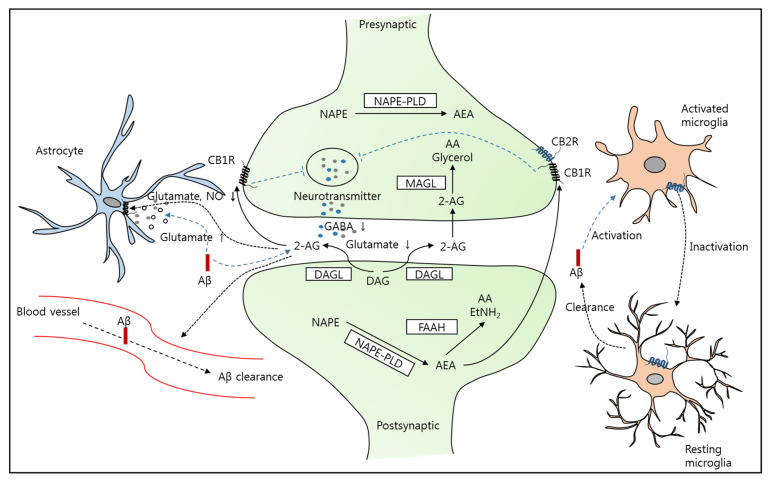
Schematic representation of the modulation of the endocannabinoid system (ECS) involved in AD pathology. In the central nervous system, the main components of the ECS (such as AEA and 2-AG) are diffusely distributed in both the presynaptic and postsynaptic compartments. AEA is synthesized through NAPE–PLD and degraded by the enzyme FAAH, whereas 2-AG is synthesized through DAGL in the postsynaptic terminal and degraded by MAGL. The release of AEA and 2-AG into the synaptic cleft stimulates CB1/CB2 receptors on presynaptic neurons to inhibit the release of neurotransmitters such as glutamate or GABA, leading to synaptic dysfunction. Aβ enhances 2-AG signaling, leading to the failure of neurotransmission. In addition, Aβ stimulates both astrocytes and microglia and accelerates neuronal damage through glutamate-mediated excitotoxicity and neuroinflammation. Modulation of the ECS has been suggested to reverse several pathological symptoms of AD. For example, the pharmacological elevation of 2-AG/AEA activates CB1 receptors on astrocytes to reduce the release of glutamate and NO and facilitates Aβ clearance through blood vessels. Moreover, activation of CB2 receptors on microglia results in the recovery of the anti-inflammatory phenotype of microglia to increase Aβ clearance. The blue dotted arrows indicate the signaling pathways induced by Aβ.

The ECS participates in a wide range of physiological and cognitive processes such as embryonic and postnatal development [27], appetite [28], pain sensation [29], emotion [30], learning and memory [30,31], and the pharmacological effects of cannabis [32,33]. The ECS involves endocannabinoids (the best characterized are anandamide [AEA] and 2-arachidonoylglycerol [2-AG]), enzymes required for their synthesis and degradation (fatty acid amide hydrolase [FAAH], monoglyceride lipase [MAGL] and diacylglycerol lipase [DAGL]), and cannabinoid receptors (the well-identified receptors are G protein-coupled CB1 and CB2 receptors) (Figure 2). Postmortem analysis revealed alterations in several components of the ECS in human AD brains. The level of CB1 receptors was significantly decreased, whereas the level of CB2 receptors was markedly increased in the frontal or parahippocampal cortex of patients with AD, probably in a time-dependent manner [34,35,36,37]. Furthermore, increased expression of CB2 receptors was selectively abundant in the plaques and surrounding areas, indicating a correlation between the ECS and plaque deposition, although the levels of CB1 and CB2 receptors were not correlated with cognitive status [34,35]. In addition, the expression of FAAH, DAGL, and MAGL was increased in the brains of patients with AD [34,38,39]. These results imply that alterations in the ECS may be involved in the pathological development of AD. Moreover, clinical trials have reported that cannabinoids might be useful for the treatment of anorexia and behavioral symptoms in patients with AD or dementia [40,41]. Therefore, considering the modulatory effects of cannabinoids on the ECS, cannabinoids may be good candidates for treating AD [42] (Figure 2).

CB1 receptors mediating the psychoactivity of cannabinoids are concentrated in the central nervous system (CNS) neurons and are coupled with decreased glutamate or γ-aminobutyric acid (GABA) transmission through the inhibition of adenylate cyclase activity and reduction in Ca^2+^ influx. On the contrary, CB2 receptors that regulate the release of cytokines are mostly located in microglia and peripheral immune cells [43,44]. However, this has been challenged by recent accumulating evidence that demonstrated the neuronal localization of CB2 receptors [37,45]. Endocannabinoids AEA and 2-AG are known to suppress presynaptic neurotransmitter release through the activation of presynaptic CB1 and CB2 receptors [44]. Animals injected with Aβ_1-42_ fragment in the deep frontal cortex showed increased levels of 2-AG but not AEA in the hippocampus and impaired memory retention in the passive avoidance test [46]. Enhanced 2-AG signaling in AD could contribute to synaptic failure, probably because of prolonged depolarization-induced suppression of inhibition in the hippocampus [38]. However, application of VDM-11, a potent cannabinoid reuptake inhibitor that increases both AEA and 2-AG levels by inhibiting endocannabinoid cellular reuptake in the hippocampus, can reverse neuronal damage and memory deficits, indicating the concomitant neuroprotective role of endocannabinoids [46], although the mechanisms have not been fully elucidated. Aβ-induced neuroinflammation can accelerate neuronal death through connexin 43 hemichannel-dependent release of glutamate and ATP from astrocytes, which can be blocked by the activation of CB1 and CB2 receptors with endocannabinoids (AEA and 2-AG) or synthetic cannabinoids (WIN-55 and 212-2) [47]. AEA and 2-AG have specific neuroprotective functions. For example, only AEA, not 2-AG, augments Aβ-impaired Notch-1 signaling, which is associated with compromised neurogenesis and cognitive function. Particularly, the effect of AEA on Notch-1 signaling is in parallel with reduced APP processing by γ-secretase [48]. This result suggests the potential role of endocannabinoids in reducing Aβ generation. Conversely, elevated 2-AG facilitates Aβ clearance across the blood brain barrier through CB1 and CB2 receptors [49] (Figure 2).

Moreover, two cannabinoid receptors are independently involved in mechanisms against AD pathology. This is probably because of the distinct expression patterns of cannabinoid receptors. Many studies have suggested that cannabinoids protect neurons from Aβ-induced neurotoxicity, mainly through CB1 receptors. In addition, endocannabinoids AEA and noladin ether (2-Arachidonyl glyceryl ether) prevent Aβ-induced cell death through CB1 receptors and the downstream mitogen-activated protein kinase (MAPK) pathway [50]. Selective activation of CB1 receptors can inhibit astroglial-derived nitric oxide (NO) production and blunt tau protein hyperphosphorylation in a coculture model [51]. The neuroprotective role of CB1 receptor activation through inhibition of Aβ-induced NO production accompanied by reduced gliosis and improved spatial memory in the eight-arm radial maze was observed in an in vivo study [52]. Consistently, CB1 receptor agonists reversed Aβ-induced electrophysiological changes such as reduced firing frequency, increased spike frequency adaptation ratio, and decreased afterhyperpolarization amplitude [53]. In addition, CB1 receptors depleted in a mouse model generated by crossing CB1 receptor knockout mice with APP23 mice, a model of AD, aggravating spatial memory impairments. However, paradoxical results such as reduced APP processing and amyloid plaque load were observed in CB1 receptor^−/−^/APP23 mice [54]. These discrepant results may be explained by the complex role of CB1 receptors in mouse development [55].

In contrast, a growing body of evidence indicates that CB2 receptors are potential therapeutic targets for AD treatment by blocking microglial activation [56]. CB2 receptors are abundantly expressed in activated microglia, which migrate around senile plaques [34,35,57]. CB2 receptor-selective agonists can counteract Aβ-induced microglial activation and subsequently facilitate neuronal survival in culture [57]. The improvement of cognitive impairment by the activation of CB2 receptors that showed increased expression in AD can be interpreted by the recovery of CB2 receptor function, which was interrupted by enhanced nitration under pathological conditions [57]. In transgenic APP/PS1 mice, activation of CB2 receptors can effectively relieve microglial activation, promote the conversion of proinflammatory microglia to anti-inflammatory microglia, and restore dendritic complexity in the cortex and thereby, improve novel object recognition memory, albeit with no effect on plaque deposition and spatial memory [58]. However, although genetic deletion of the CB2 receptor reduces microglia-mediated neuroinflammation and ameliorated spatial memory deficits at 14 months of age, no improvement is observed at a younger age [59,60]. Recent studies have corroborated the neuronal localization of CB2 receptors. Consistent with the aforementioned studies on microglia, both genetic ablation and pharmacological activation of the CB2 receptor in neurons can have neuroprotective roles against AD-related neurodegeneration [37,45]. Therefore, the role of CB2 receptor signaling in AD pathology remains unclear. Either aggravated Aβ deposition or decreased plaque levels were found in AD mice with CB2 receptor deficiency [59,60,61]. Nevertheless, the pharmacological activation of the CB2 receptor consistently benefits Aβ clearance [56,62,63]. However, the conclusion that the genetic modulation of CB2 receptors has a neuroprotective role should be more carefully and deeply corroborated. Collectively, cannabinoids might effectively alleviate both amyloid and tau pathologies in AD by modulating CB1 and CB2 receptors.

## 3. Alzheimer’s Disease and Cannabidiol (CBD)

Phytocannabinoids are natural cannabinoids found in cannabis plants. More than 400 different compounds are present in the cannabis plant, of which delta-9-tetrahydrocannabinol (Δ9-THC) and cannabidiol (CBD) are the most well-known compounds. Sativex, a mixture of THC and CBD, has been approved for the treatment of multiple sclerosis by the US FDA. Administration of Sativex at early symptomatic stages induces a reduction in soluble Aβ42 levels and improves cognitive deficits in APP/PS1 mice [64]. In addition, in a human tau transgenic mouse model, administration of Sativex resulted in reduced phosphorylated tau, GSK3 expression, and levels of Aβ oligomers [64,65]. Hence, the components of the ECS are believed to be the potential targets of phytocannabinoids to protect against AD pathology. However, modulation of the ECS in response to phytocannabinoids is much more complicated. Microarray studies have revealed that the Sativex induces complex differential gene expression profile in APP/PS1 mice containing 187 upregulated and 136 downregulated genes [64], which include alterations in genes associated with inflammatory, antioxidant, intracellular stress, Wnt signaling, as well as mitochondrial function and autophagy [64]. In addition, THC can directly bind to AChE through a peripheral anionic site, which is also involved in amyloidogenesis, thereby inhibiting the degradation of acetylcholine and AChE-induced Aβ aggregation [66]. Overall, the neuroprotective function of phytocannabinoids not only refers to various consequences of modulating the ECS but also includes the regulation of multiple cannabinoid receptor-independent signaling pathways.

Although THC may have a complex beneficial role against AD pathologies such as stimulation of CB1 receptors, the concomitant psychotropic effects of THC pose a problem. However, CBD exerts multiple neuroprotective functions through the modulation of various receptors but is devoid of psychotropic effects and is well-tolerated [67,68,69]. Therefore, the neuroprotective and nonpsychoactive properties of CBD have attracted increasing attention for its therapeutic potential, especially in AD.

Early studies have suggested that CBD has a very low affinity for both CB1 and CB2 receptors and acts as a weak antagonist in the micromolar range [69,70]. Interestingly, CBD was identified as an inverse agonist of CB2 receptors to effectively antagonize the effects of cannabinoid receptor agonists [71]. CB2 receptor inverse agonists can inhibit immune cell migration; hence, CBD may share a similar mechanism for its anti-inflammatory property [72]. In addition, CBD might inhibit the enzymatic activity of FAAH and increase the levels of AEA and 2-AG, thereby suppressing excessive glutamatergic transmission in AD pathology [69,73]. Apart from classic CB1 and CB2 receptors, G protein-coupled receptor 55 (GPR55) is considered an additional cannabinoid receptor despite the lack of homology in amino acid sequences [68,74]. GPR55 is a G_α_-coupled receptor distinct from G_i_-coupled CB1 and CB2 receptors. The activation of GPR55 contributes to elevated intracellular calcium and neuronal excitability, thereby modulating cellular proliferation and migration and hippocampal release of glutamate [75,76], which might be involved in inflammation and cognitive processes.

**Figure 3:**
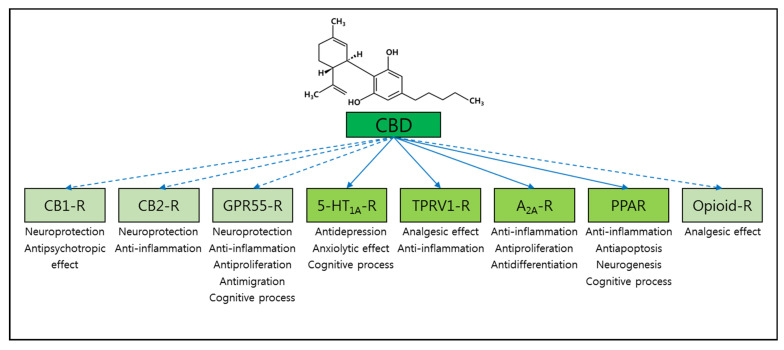
Potential biological targets of CBD in AD treatment. Based on the property of each potential receptor target of CBD and well-known mechanism studies of CBD-mediated therapeutic functions, several potential biological targets of CBD involved in AD treatment have been proposed, including CB1, CB2, GPR55, 5-HT_1A_, TPRV1, A_2A_, PPARs, and opioid receptors. Black arrows indicate receptors activated by CBD, whereas dotted arrows indicate receptors inhibited by CBD. The therapeutic roles of CBD mediated by each receptor are listed underneath to provide insights for CBD application in AD treatment.

**Figure 4:**
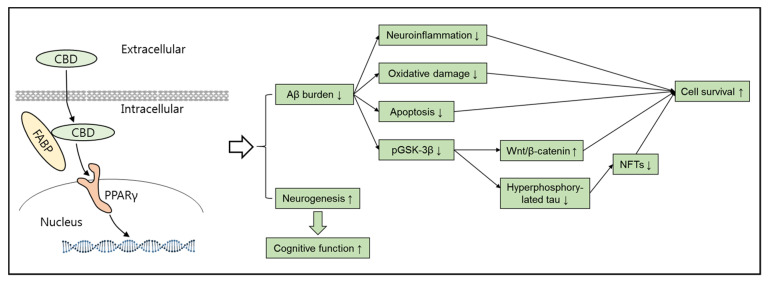
Summary of the main findings illustrating the neuroprotective functions of CBD against AD pathology. CBD can be translocated into the nucleus by FABPs and activate nuclear PPARγ to induce gene expression, resulting in a series of improvements against Aβ-induced aberrant changes in AD, such as reduced neuroinflammation, oxidative changes, and apoptosis. In addition, CBD can contribute to the inhibition of hyperphosphorelated tau and activation of Wnt/β-catenin signaling by recovering Aβ-induced pGSK-3β. Finally, the CBD-mediated therapeutic changes facilitate neuronal survival. In addition, CBD enhances neurogenesis in AD. Overall, both CBD-mediated neuroprotection against Aβ-induced pathologies and CBD-mediated increase in neurogenesis might contribute to the improved cognitive function observed in AD.

Meanwhile, it is important to highlight that CBD activates a wide spectrum of receptors such as serotonin receptors, vanilloid receptors, adenosine receptors, peroxisome proliferator-activated receptors (PPARs), opioid receptors, and dopamine receptors [73,77,78] (Figure 3). Hence, CBD has interesting therapeutic potential beyond the presumptive action of cannabinoid receptors. For example, it has already been demonstrated that the antidepressant or anxiolytic effects induced by CBD is predominantly mediated by the activation of 5-HT_1A_ receptors [77] and transient receptor potential vanilloid 1 (TRPV1) is an important molecular target for CBD treatment in various models of pain and inflammation [78]. Furthermore, previous studies have suggested that low levels of 5-HT are well associated with impaired memory [79] and activation of 5-HT_1A_ by CBD ameliorates cognitive impairments in bile duct ligated mice [80]. In line with these observations, stimulation of TRPV1 expressed in microglia blunted central inflammation in multiple sclerosis in a model of experimental autoimmune encephalomyelitis, suggesting the potential therapeutic use of CBD in neurological diseases through anti-inflammatory properties [81,82]. In fact, anti-inflammatory signaling mediated by CBD is very complex and involves enhanced activation of A_2A_ receptors or PPARs [83,84]. Nevertheless, all these receptors may interact with each other, rather than work independently, in the context of inflammation. For example, a recent study suggested that blockade of A_2A_ receptors may potentiate CB2 receptor-mediated signaling within an A_2A_-CB_2_ receptor heteromer context [85]. PPARs regulate many target genes and signaling cascades and initiate various physiological responses such as anti-inflammatory activity, cell proliferation, and differentiation [86]. CBD can be delivered to the nucleus by fatty acid-binding proteins and then CBD directly binds to PPAR, resulting in the activation of PPAR and numerous PPAR-mediated changes [84,87]. Consistently, PPARγ has been identified as the key regulator of the neuroprotective mechanisms of CBD against AD progression [88] (Figure 4). In addition, CBD has been reported to be an allosteric modulator of μ- and δ-opioid receptors [89]. This property may contribute to the latent possibility of CBD treatment in AD because of the accelerated Aβ pathology through a complex formed by the δ-opioid receptor with β- and γ-secretases [90]. Overall, CBD seems to be an attractive candidate that meets the requirement for a novel preventive AD therapy, which requires a multifunctional drug targeting several AD pathologies simultaneously.

Previous studies have observed a promising effect of CBD on improving cognitive impairment in AD mice. Pretreatment of hippocampal slices with CBD attenuated Aβ-mediated deficits in long-term potentiation, a major cellular mechanism underlying memory formation, through PPARγ modulation [91]. In mice, exogenous injection of fibrillar Aβ in the brain induces spatial memory deficits that can be reversed by intraperitoneal CBD administration [92]; however, acute injection of exogenous Aβ cannot mimic the real progressive Aβ accumulation that occurs in patients with AD. In transgenic APP/PS1 mice, deficits in social and objective recognition memory have been developed, similar to the facial recognition impairments observed in patients with AD, and chronic CBD treatment from the early symptomatic stage neutralized these cognitive deficits without any changes in anxiety and associative memory at 7–10 months of age [26,64,93,94]. It is noteworthy that the behavioral changes were not completely consistent across studies, probably due to differences in the mouse models used and CBD treatment scheme [95].

The amyloid cascade is regarded as the key factor that contributes to cognitive impairment in AD. An in vitro study showed that CBD treatment counteracted the elevated expression of APP and Aβ in APP-transfected human neuroblastoma SH-SY5Y^APP+^ cells in a dose-dependent manner [96]. This may be because of the CBD-mediated increase in APP ubiquitination with the involvement of PPARγ [96]. However, such an effect was barely observed in CBD-treated APP/PS1 mice [64,93,97]. Therefore, these results indicate that CBD has a modest pharmacological role in suppressing aberrant APP processing, which is only observed under rigorous simple experimental conditions. On the contrary, CBD inhibits hyperphosphorylation of tau in Aβ-stimulated PC12 neuronal cells in a dose-dependent manner [98]. GSK-3β, a multifunctional serine/threonine kinase, is a well-known upstream mediator of tau hyperphosphorylation [99]. As expected, Aβ treatment results in the formation of phosphorylated GSK-3β, whereas CBD reverses this progress and changes downstream of GSK-3β [98]. However, it does not seem convincing enough because this result has not yet been verified in AD animal models.

Although evidence has shown a slight effect of CBD on Aβ and tau pathology in vitro, CBD-mediated improvement in cognitive behavior is associated with the neuroprotective role of CBD against Aβ-induced cytotoxicity. It has been reported that in PC12 cells, CBD decreases Aβ-stimulated ROS and NO production, along with p38 MAPK and NF–κB, which is a redox-sensitive transcription factor that is activated under oxidative and inflammatory conditions [100,101]. Moreover, an increased expression of p50 and p65, the components of NF–κB, parallel to upregulated GFAP, S100, iNOS, and inflammatory factors was observed in Aβ-treated cultured astrocytes [102]. CBD can blunt Aβ-induced oxidative stress and neuroinflammation in astrocytes through PPARγ [102]. Meanwhile, Aβ-induced neuroinflammation and oxidative stress were associated with increased cell death and CBD treatment before Aβ treatment or to APP-transfected neuroblastoma cells improved cell survival [96,100]. In addition, CBD-treated SH-SY5Y^APP+^ cells showed a smaller number of apoptotic cells. The PPARγ antagonist (GW9662) reversed the inhibition of apoptotic events by CBD, indicating that PPARγ contributes to neuronal survival and mediates the effect of CBD on Aβ-induced apoptosis [96]. It has been well documented that Wnt/β-catenin signaling inhibits neuronal apoptosis and improves neuronal survival by inhibiting GSK3β [73,103]. CBD can inhibit Aβ-induced activation of GSK-3β, suggesting a possible mechanism [98,104]. Furthermore, in a rat model of AD established by the intrahippocampal injection of fibrillar Aβ, CBD promoted neuronal survival and increased DCX-labeled cells, indicating an increase in neurogenesis [102] (Figure 4).

Overall, recent studies have provided preliminary evidence for the therapeutic potential of CBD against AD with its anti-inflammatory, antioxidative, antiapoptotic, and neurogenic properties. Nevertheless, several concerns related to the mechanisms of CBD are required to be addressed in further investigations. For example, modulation of the ECS components has been reported to achieve benefits against AD pathology, but the mechanism through which the cannabinoid receptor directly regulates the neuroprotective roles of CBD against AD pathology has not been studied. In addition, it remains unclear whether the pharmacological effects of CBD observed in AD therapy are associated with receptors other than PPARγ. Moreover, whether excitatory neurons and inhibitory neurons differently respond to CBD in AD pathological conditions should be investigated in the future.

## 4. Conclusions

AD, the most common neurodegenerative disease, accounts for the most prominent form of dementia. Unfortunately, current therapies only provide limited symptomatic efficacy. The ECS regulates various pathological processes related to AD. CBD, a cannabinoid, might serve as a promising candidate with the advantage of targeting both cannabinoid and noncannabinoid receptors. Many studies reviewed here suggest that CBD can provide symptomatic relief or even arrest AD progression through anti-inflammatory, antioxidative, and neurogenic effects. Moreover, CBD is a well-known inverse agonist of cannabinoid receptors, the modulation of which is reported to be beneficial for preventing AD pathology. Further studies are warranted to optimize CBD treatment for therapeutic dosage and timing. It has been corroborated that PPARγ is critical for the multifunctional roles of CBD but whether other receptors or signaling pathways participate is elusive. CBD upregulates the mRNA levels of genes encoding heat shock proteins and ubiquitin-conjugating enzymes, which are considered important modulators of autophagy [105]. Autophagy is impaired in AD and can be affected by combinations of THC and CBD [14,65]; therefore, it is rational to hypothesize that CBD may modulate impaired autophagy in AD. A recent study assessed autophagic changes induced by chronic CBD treatment in 6-month-old APP/PS1 mice and confirmed the pharmacological effects of CBD on autophagy in AD [97]. However, more evidence is required to illustrate the facticity of CBD-induced autophagic regulation and mechanism of autophagy modulation by CBD in AD pathology. Availability of these data will facilitate the characterization of the pharmacological effects of CBD in depth and accelerate the translation of this preclinical study into clinical settings.

## Data Availability

Not applicable.

## Abbreviation List

2-AG	2-Arachidonyl Glycerol
5-HT	5-Hydroxytryptamine
AChE	Acetylcholinesterase
AD	Alzheimer’s Disease
AEA	Anandamide
APP	Amyloid Precursor Protein
ATP	Adenosine Triphosphate
Aβ	Amyloid β
CB1R	Cannabinoid Receptor Type 1
CB2R	Cannabinoid Receptor Type 2
CBD	Cannabidiol
DAGL	Diacylglycerol Lipase
DCX	Doublecortin
ECS	Endocannabinoid System
FAAH	Fatty Acid Amide Hydrolase
GABA	γ-Aminobutyric Acid
GSK-3β	Glycogen Synthase Kinase-3β
LTP	Long-term Potentiation
MAGL	Monoglyceride Lipase
MAPK	Mitogen-Activated Protein Kinase
mGluR5	Metabotropic Glutamate Receptor 5
NAPE-PLD	*N*-Acyl Phosphatidyl Ethanolamine Specific Phospholipase D
NMDA	*N*-Methyl-D-Aspartate
NO	Nitric Oxide
PIP2	Phosphatidylinositol-4,5-Bisphosphate
PPARγ	Peroxisome Proliferator-Activated Receptor Gamma
PS1	Presenilin 1
ROS	Reactive Oxygen Species
THC	Δ9-Tetrahydro Cannabinol
TRPV1	Transient Receptor Potential Vanilloid 1
